# "Triple Trouble" in A Case of Mass Lesion

**DOI:** 10.3779/j.issn.1009-3419.2020.102.43

**Published:** 2020-12-20

**Authors:** K. KOWSHIKREDDY, Ranganadin PAJANIVEL, Ramalingam BASKARAN, Selvaraj KARTHIKEYAN, C.S. PRABHU

**Affiliations:** 1 Department of Pulmonary Medicine, Mahatma Gandhi Medical College and Research Institute, Sri Balaji Vidyapeeth University, Pondicherry 607402, India; 2 Department of Cardiology, Mahatma Gandhi Medical College and Research Institute, Sri Balaji Vidyapeeth University, Pondicherry 607402, India; 3 Department of Radiology, Mahatma Gandhi Medical College and Research Institute, Sri Balaji Vidyapeeth University, Pondicherry 607402, India

**Keywords:** Lung neoplasms, Intra-cardiac metastasis, Pulmonary tuberculosis

## Abstract

Bronchogenic carcinoma, the commonest lung tumor occurs more frequently in the elderly with typical symptoms of cough, haemoptysis, weight loss, dyspnoea or chest pain. These symptoms mimic common respiratory infections in en-demic areas like pulmonary tuberculosis. Also, metastasis at presentation itself is common, the favoured sites being liver, contra-lateral lungs, bones, brain, *etc*., although unusual and rare sites like heart also are known. We herein report a rare association of both carcinoma with active pulmonary tuberculosis in the same lobe associated with intracardiac metastasis. Very few cases have been published describing lung carcinoma with intracardiac metastasis. We hope the documentation of this rare case will shed further light into the subject area and improve clinical education.

## Introduction

Lung cancer is one of the most common malignancies in the world, with an incidence of 1.8 million cases and 1.6 million deaths, annually, according to the World Health Organization's most recent cancer report. The 5-year survival rate is poor at approximately 15%^[1]^ probably due to metastasis or advanced stage at presentation in most cases.

Despite the number of cases, metastasis to other organs such as heart are very rarely documented. The primary reason being that the absence of early symptoms makes a clinical diagnosis of cardiac metastasis difficult^[2]^. Furthermore, even when present, such symptoms are often masked by the clinical features of advanced lung cancer^[3]^.

The incidence of lung cancer is greater in patients with pulmonary tuberculosis (5%-6.4%) than the general population^[4-6]^.

## Case history

A 70-year male presented to our tertiary care hospital with complaints of dry cough, breathlessness on exertion, dysphonia, weight loss, fatigue, and loss of appetite for 2 months and 15 days history of giddiness. He is a current smoker with pack-years of forty. He doesn't have a significant past history or co-morbidities.

On general examination, he was conscious, oriented, and tachypnoeic. Pulse was 75 beats per minute, irregularly irregular with variable volume. Oxygen saturation was 95%. Clubbing-Present. On chest auscultation, bilateral normal breath sounds with diminished intensity was heard. On cardiac auscultation, S1 was variable, S2 heard, no murmur.

**Figure 1:**
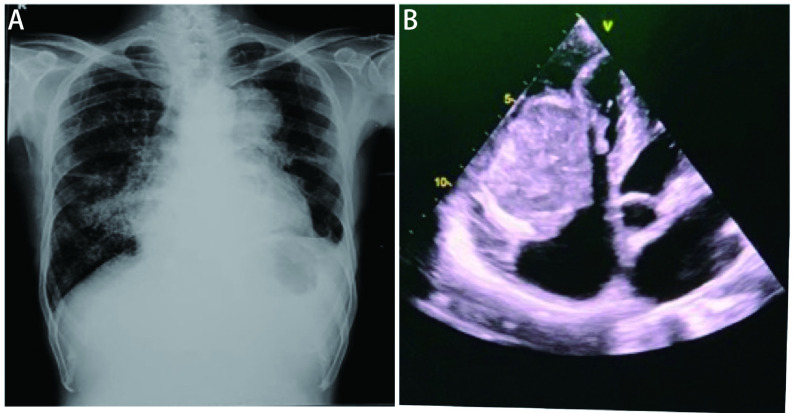
CXR and ultrasound images. A: CXR shows left upper zone homogenous opacity with left CP angle blunting and left diaphragm elevation, nodular opacities are seen in right middle and lower zone, cardiomegaly with prominent right cardiac borders and right fissure thickening; B: A mixed echoic mass measuring 4 cm×6 cm in RV in flow just below the tricuspid valve without causing significant turbulence across tricuspid valve. CXR: chest radiograph.

Next, we took electrocardiogram (ECG), in view of suspected arrhythmia and it showed atrial fibrillation (AF). Further, a chest X-ray was done it showed left upper para-mediastinal homogenous opacity with left costo-phrenic (CP) angle blunting, elevated left dome of the diaphragm, nodular opacities in the right middle and lower zone, cardiomegaly with prominent right cardiac border (Fig 1A).

Hence, we proceeded with 2D echo to find out the cause for AF. It showed a mixed echoic mass measuring 4 cm×6 cm, occupying right ventricle (RV) inflow just below the tricuspid valve without causing significant turbulence across the valve; RV dysfunction with mild tricuspid and mitral regurgitation and pericardial effusion (Fig 1B). In view of AF, caused by the intracardiac mass, he was started on low molecular weight heparin and anti-arrhythmic.

**Figure 2:**
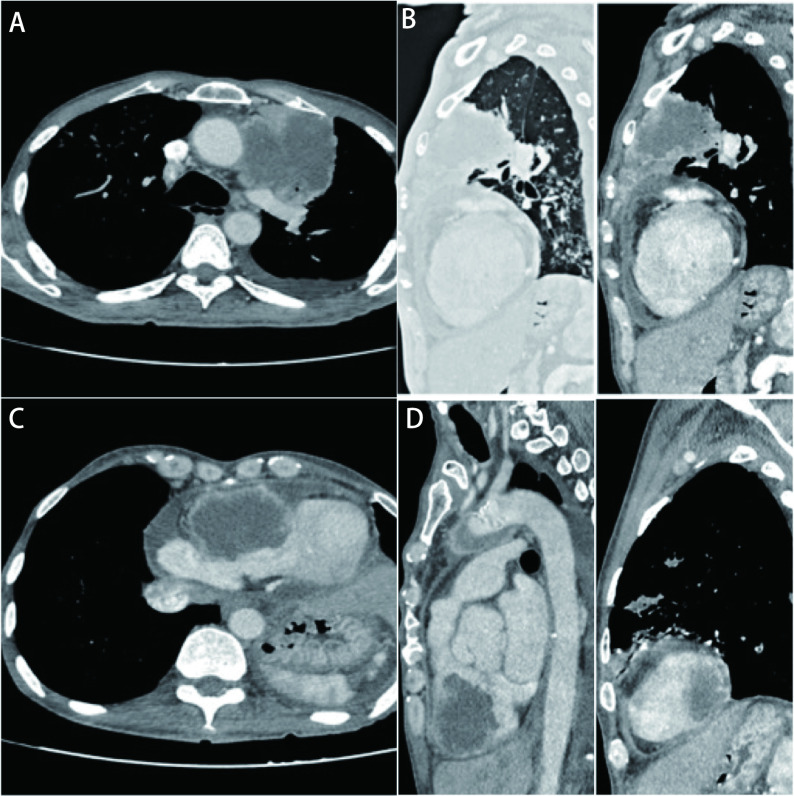
CT images. A: Axial cut showing a well-defined heterogeneously enhancing lesion with irregular margins noted in left upper lobe and bilateral multiple nodules and minimal pleural effusion on left side; B: Sagittal cut showing a well-defined heterogeneously enhancing lesion with irregular margins noted in left upper lobe; C: Axial cut showing a heterogeneous opacity in right ventricle of heart, exophytic and completely intraluminal with moderate pericardial effusion; D: Sagittal cut showing a heterogeneous opacity in right ventricle of heart, exophytic and completely intraluminal and a small heterogeneous opacity along lateral left ventricular wall. CT: computed tomography.

Contrast-enhanced computed tomography (CECT) of thorax was done to evaluate the lung lesion, a well-defined heterogeneously enhancing lesion measuring 7.3 cm×6.3 cm×6.5 cm with irregular margins in the left upper lobe and it infiltrates the mediastinal pleura. Multiple nodules were noted bilaterally. Minimal pleural effusion was seen on the left side (Fig 2).

The cardiac image in CECT showed a heterogeneous lesion with similar density and enhancement as the lung lesion measuring 6.6 cm×6.6 cm×5.9 cm in the right ventricle which is completely intraluminal. Another small similar appearing lesion noted along the lateral left ventricular wall measuring 2.1 cm×2 cm. Diffuse pericardial thickening with associated mild to moderate pericardial effusion (Fig 2).

**Figure 3:**
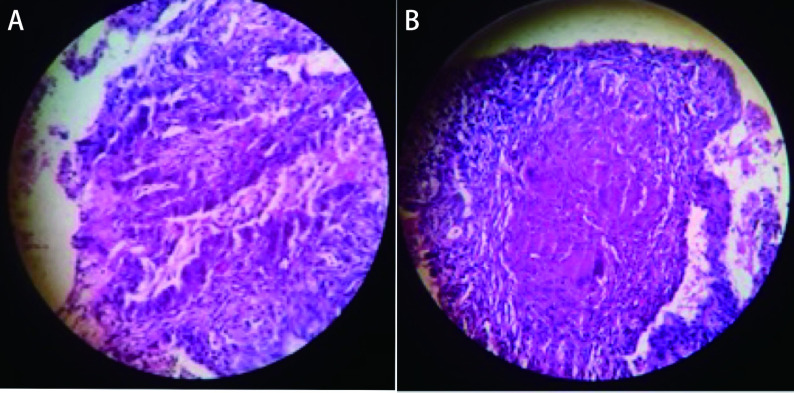
Pathological pictures. A: A tumor arranged in glands and acini which have moderate degree of nuclear pleomorphism, hyperchromatic nuclei with scanty cytoplasm and vacuolations; B: HPE showing tissue with alveoli and pneumocyte hyperplasia with interstium showing epithelioid granulomas with caseous necrosis.

We decided to evaluate the lung mass, hence CT guided trucut biopsy of left upper lobe mass lesion was performed. Histopathological examination showed a tumor arranged in a glandular and acinar pattern. Individual cells show a moderate degree of nuclear pleomorphism, hyperchromatic nuclei with scanty cytoplasm, and vacuolations (Fig 3A). Surprisingly, there were other foci showing tissue with alveolar and pneumocyte hyperplasia and interstitium showing epithelioid granulomas with caseous necrosis (Fig 3B). These features suggestive of adenocarcinoma lung with associated tuberculosis. Immunohistochemistry was done and showed positive for thyroid transcription factor 1 (TTF-1).

To confirm the microbiological diagnosis of pulmonary TB, sputum for acid fast bacilli smear and GeneXpert for *Mycobacterium Tuberculosis* was planned but couldn't be done as the patient was not expectorating.

We planned for cardiac magnetic resonance imaging (MRI) to study the further details of cardiac mass but couldn't able to execute due to unstable hemodynamic condition. The patient's condition got deteriorated and became critical and expired before proceeding to whole-body positron emission tomography-CT (PET-CT) imaging.

The final diagnosis made was adenocarcinoma of the lung with pulmonary tuberculosis and AF secondary to intracardiac metastasis.

## Discussion

Carcinoma lung is the most common type of cancer in the world and the most common cause of cancer‐related mortality^[3]^ metastasis is the most common cause of death from lung cancer, the common sites being lymph nodes, liver, brain, bone and adrenal glands in decreasing order of frequency^[7]^.

Cardiac metastases occur predominantly between the sixth and eighth decade, and there is no gender predisposition^[8]^. The rate of metastasis of lung cancer to heart varies with each histopathological subtype: adenocarcinoma metastasizes to the heart in 26% of cases, squamous cell carcinoma in 23.4%, undifferentiated carcinoma in 21.2% and bronchoalveolar carcinoma in 17.4%^[8]^.

Epicardium/pericardium is the commonest site of cardiac metastasis, whereas myocardial, endocardial, intracavitary, and valvular metastasis are rare^[9]^. 80% of metastasis occur to the right chambers, with the right atrium being the most affected, as a result of slower flow, low intracavitary pressure, lighter contractile strength and their filtering role in the pulmonary circulation^[10]^.

The various mechanisms of cardiac metastases include lymphatic spread (most common), direct extension from the adjacent viscera, hematogenous spread, and transvenous extension^[11]^.

Bronchogenic carcinoma may involve the heart and pericardium by direct extension or by a combination of lymphatic and hematogenous dissemination. Involvement of the heart and pericardium is usually found at autopsy and direct extension^[12]^ also represent stage IV disease in the American Thoracic Society's tumor-node-metastasis (TNM) staging system for bronchogenic carcinoma.

Pericardial metastases occur late in the course of a neoplasm. Lung cancer, the most frequent source, accounts for about one-third of the cases, followed by breast cancer.

In majority of the patients (approximately 90%), cardiac metastasis is silent and diagnosed only on autopsy^[11]^. The clinical features are extremely variable and depend on the anatomic location, size of the tumor, and any invasion of adjacent tissues. Clinical manifestations are caused by direct obstruction of cardiac or valve function, interruption of coronary flow, interference with electrophysiology of contraction, and pericardial effusion. Intramural tumours can cause arrhythmias, obstruction of the right or left outflow tract, and compression of the cardiac chambers. Intracardiac tumors cause clinical features of right or left‐sided heart failure^[11]^.

The introduction and widespread use of sophisticated imaging modalities have resulted in a significant increase in the incidental detection of metastasis.

Echocardiography is the investigation of choice for the diagnosis. Trans-oesophageal echo offers better visualization of the atria and great vessels as compared to other modalities. CT and MRI are also useful tools that provide information on location, morphological features, extent, presence of local invasion, and mediastinal or pulmonary involvement^[12]^.

The relationship between pulmonary TB and bronchogenic carcinoma has been viewed in the following ways: (1) As one of cause and effect *i.e*., scar tissue plays an important causative role in the development of lung cancer^[13, 14]^; (2) The reactivation of TB by carcinoma^[15, 16]^. This association may also be related to increased susceptibility to opportunistic infections, which can lead to the reactivation of TB in cancer patients; (3) As coincidental.

There are two potential mechanisms in the pathogenesis of this association. First, that the carcinoma invaded the existing tuberculous lesions resulting in activation. Second, that the tuberculosis relapsed intrinsically upon contact with carcinomatous lesions. However, in this case, it cannot be denied that the carcinoma invaded the tuberculous lesions in the margin of the hilum carcinoma, resulting in its activation, and spread to the distal lesion of the lung^[15]^.

Due to its close proximity, cardiac metastasis from advanced lung cancer may get overlooked as the clinical focus is directed toward the primary malignancy. Most of the lung cancers usually present in advanced stages, leading to poor prognosis. The presence of metastasis, especially intracardiac, carries the worst prognosis. Coexistent lung carcinoma with pulmonary tuberculosis makes clinical management challenging and is associated with a high mortality rate.

To the best of our research, a similar case has not been reported in the literature. Considering as a challenging case to every physician, we hope the documentation of this rare case will shed further light into the subject area and improve clinical education.
